# Bayesian Mendelian randomization with an interval causal null hypothesis: ternary decision rules and loss function calibration

**DOI:** 10.1186/s12874-023-02067-4

**Published:** 2024-01-27

**Authors:** Linyi Zou, Teresa Fazia, Hui Guo, Carlo Berzuini

**Affiliations:** 1 Centre for Biostatistics, School of Health Sciences, The University of Manchester, Jean McFarlane Building, Oxford Road, Manchester, M13 9PL UK; 2 Department of Brain and Behavioural Sciences, University of Pavia, Pavia, 27100 Italy

**Keywords:** Mendelian randomization, Region of practical equivalence, Interval null hypothesis, Ternary decision logic, Loss function calibration, Juvenile myocardial infarction

## Abstract

We enhance the Bayesian Mendelian Randomization (MR) framework of Berzuini et al. (Biostatistics 21(1):86–101, 2018) by allowing for interval null causal hypotheses, where values of the causal effect parameter that fall within a user-specified interval of “practical equivalence” (ROPE) (Kruschke, Adv Methods Pract Psychol Sci 1(2):270–80, 2018) are regarded as equivalent to “no effect”. We motivate this move in the context of MR analysis. In this approach, the decision over the hypothesis test is taken on the basis of the Bayesian posterior odds for the causal effect parameter falling within the ROPE. We allow the causal effect parameter to have a mixture prior, with components corresponding to the null and the alternative hypothesis. Inference is performed via Markov chain Monte Carlo (MCMC) methods. We speed up the calculations by fitting to the data a simpler model than the intended, "true", one. We recover a set of samples from the “true” posterior distribution by weighted importance resampling of the MCMC-generated samples. From the final samples we obtain a simulation consistent estimate of the desired posterior odds, and ultimately of the Bayes factor for the interval-valued null hypothesis, $H_0$, vs $H_1$. In those situations where the posterior odds is neither large nor small enough, we allow for an *uncertain* outcome of the test decision, thereby moving to a ternary decision logic. Finally, we present an approach to calibration of the proposed method via loss function. We illustrate the method with the aid of a study of the causal effect of obesity on risk of juvenile myocardial infarction based on a unique prospective dataset.

## Introduction

The causal effect of an exposure on an outcome can, under certain assumptions, be assessed from observational data by using measured variation in genes as an instrumental variable. This is called a Mendelian Randomization (MR) analysis (Katan [1]; Smith and Ebrahim [2]; Lawlor et al. [3]; Jeffrey [4]). We approach MR via a parametric model of an assumed data generating process, where the unknown magnitude of the causal effect is represented by a parameter, $\beta$. At least initially, we assume $\beta$ to be a scalar. In this context, the null hypothesis $H_0$ of non-existence of the causal effect is often represented by $\beta$ taking a particular value that with no loss of generality we take to be 0. In this case we have a *point null* causal hypothesis and a diffuse alternative hypothesis $H_1: \beta \ne 0$.

It is however possible to replace the point null causal hypothesis with an *interval null* causal hypothesis $H_0: \mid \mid \beta \mid \mid \; \le T$, corresponding to the alternative hypothesis $H_1: \mid \mid \beta \mid \mid \; >T$. In this case the user must specify a positive *T* in such a way that causal effect values within $[-T, T ]$ can be regarded as practically equivalent to zero. The justification for introducing the $[-T, T]$ interval is similar to that of the *Region of Practical Equivalence* (ROPE) of Kruschke [5], and we shall therefore use “ROPE” to refer to that interval. By regarding values of the causal effect that are “too close” to 0 as equivalent to 0, the ROPE approach protects us from a problematic aspect of point null hypothesis testing, namely, that it will always reject the null as the sample size tends to infinity, due to inevitable departures of the model from “truth”. Use of the ROPE will protect us from this phenomenon by avoiding statistically significant but artefactual, and typically minuscule, estimates of the effect. These occur in MR analysis due to MR models being often a rough approximation of an underlying process, and furthermore relying on untestable assumptions. By using ROPE, we reduce the risk of an MR analysis leading to a causal discovery claim in the presence of only scant evidence in favour of the alternative, as well as the risk of the null hypothesis being accepted in spite of the data being compatible with a (possibly important) causal effect. The ROPE approach is discussed by a number of authors, including Stanton [6]; Kelter [7]; Kelter [8]; Liao et al. [9]; Linde et al. [10] and Stevens and Hagar [11].

Not only are we standing in favour of interval null (as opposed to point null) hypotheses in MR, but also of a Bayesian (as opposed to a frequentist) approach to hypothesis testing, main reasons being its ability to use information about the relative levels of evidence for both the null and alternative hypotheses (Berger and Sellke [12]) and its freedom from asymptotics. Motivated by the above considerations, we combine the strengths of Bayesian hypothesis testing with those of ROPE within the Bayesian MR framework of Berzuini et al. [13], further refined by Zou et al. [14].

We entertain a parametric model of the assumed data generating process with the causal parameter $\beta$ drawn from a mixture prior distribution. One component of which, with fixed weight $\pi _0$, represents the distribution of $\beta$ under $H_0$, which we take to be a flat distribution with no support outside the ROPE. The second component of the mixture, with fixed weight $(1-\pi _0)$, represents the distribution of $\beta$ under $H_1$, which we take to be a continuous normal distribution with mean 0. Parameter $\pi _0$ is the user-assigned prior probability for the null hypothesis. Inference computations are made by using Markov chain Monte Carlo (MCMC) methods of simulation. By combining MCMC with importance sampling, we are allowed to fit the data to a simpler (and incorrect) model, where $\beta$ has an “importance” continuous prior distribution. Weighted resampling of the output posterior samples will then recover a set of samples from the posterior of the true model. Based on the proportion of samples falling within the ROPE we calculate approximations to the posterior probabilities of $\beta$ falling inside and outside the ROPE, and from these we get a simulation-consistent estimate of the posterior odds in favour of the null and, ultimately, a simulation-consistent estimate of the Bayes factor for $H_0$ vs $H_1$, as a basis for the test decision. A formal discussion of relationships between our method and standard Bayes factor calculations is available from the Authors. As common in Bayes factor analysis, whenever the posterior odds is neither large nor small enough, indicating less-than-strong evidence in favour of either hypothesis, we declare the test decision “uncertain”, thereby moving from a binary to a ternary decision logic.

Finally, for purposes of decision rule calibration, we introduce a loss function that measures the "cost" incurred by each possible combination of a decision outcome and true hypothesis. We use this for model parameter tuning and comparison of a classical and a Bayesian approach to MR.

We illustrate the methods with the aid of a MR study of the effect of obesity on risk of juvenile myocardial infarction, on the basis of a unique set of data from patients hospitalized for myocardial infarction between 40 and 45 years of age and healthy controls.

## Methods

### Bayesian Mendelian randomization model

**Fig. 1:**
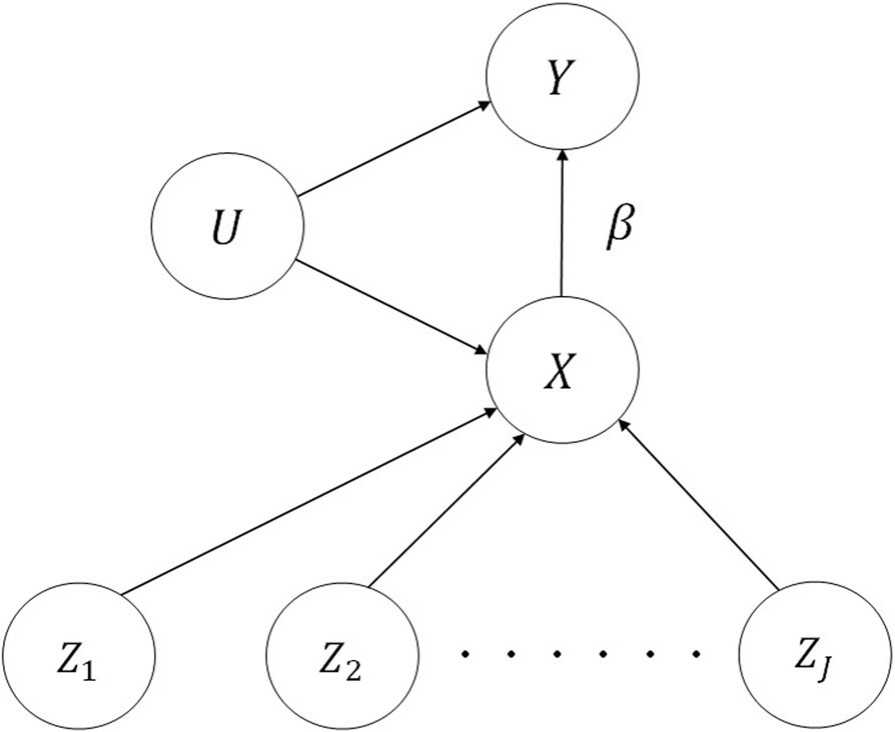
Directed acyclic graph (DAG) representation of the Mendelian randomisation model we consider throughout the paper. The strength of the $X \rightarrow Y$ arrow, corresponding to the unknown magnitude of the causal effect of interest, is represented in the model equations by the unknown parameter $\beta$

Suppose we wish to assess the putative causal effect of a scalar exposure *X* on a scalar outcome *Y*, by using information provided by a set $\textbf{Z} \equiv (Z_1, \ldots , Z_J)$ of genetic instrumental variables (IVs, or instruments), typically single nucleotide polymorphisms (SNPs). A directed acyclic graph (DAG) representation of the proposed model for this task is shown in Fig. 1.

Suppose, for the time being, that each individual in the sample comes with a completely observed set of variables $(X, Y, \textbf{Z})$. Without infringing the argument’s general validity, let *Y* be a binary variable. Let *U* denote a scalar summary of the unobserved confounders of the relationship between *X* and *Y*. Within a Bayesian framework, if we assume standardised $(\textbf{Z}, X)$ variables and linear additive dependencies (but we could consider interaction between the effects of the $\{\textbf{Z}\}$ on *X*), then a possible parametrization of the model is:

1 $$\begin{aligned} U&\sim N(0, 0.1), \end{aligned}$$

2 $$\begin{aligned} X \mid \textbf{Z},U&\sim N\left( \sum \limits _{k=1}^{J}\alpha _{k}Z_{k}+\delta _{X}U, \sigma _{X}^{2}\right) , \end{aligned}$$

3 $$\begin{aligned} Y \mid X,U&\sim \text {Bernoulli}(\text {expit}(\omega +\beta X + \delta _{Y}U)), \end{aligned}$$

where the symbol $\sim$ stands for “is distributed as” and $\text {expit}(a) \equiv \frac{e^a}{1+e^a}$. The symbol *N*(*a*, *b*) denotes normal distribution with mean *a* and variance *b*, and $\sigma _X$ is the standard deviation of an independent random perturbation of *X*. This model is identifiable except for the δ parameters being estimable only through their product. Of inferential interest is the causal effect of the exposure *X* on the outcome *Y*, as quantified by parameter $\beta$, with $\beta =0$ corresponding to the causal effect having zero magnitude. The vector parameter $\varvec{\alpha } = (\alpha _{1}, \alpha _{2}, \ldots , \alpha _{J})$ represents the strengths of the pairwise associations (not necessarily causal) between instruments and exposure. This model is adapted from Berzuini et al. [13] and Zou et al. [14].

As is common in statistics, the DAG can be interpreted as a way of coding conditional independence relationships that are implicit in the model equations. These relationships are conveniently expressed by using the conditional independence notation of Dawid [15], where $A \,\perp \!\!\!\perp \, B \mid C$ reads: “*A* is independent of *B*, given *C*”, asserting that the conditional distribution of the random variable *A*, given the value of the random variable *C*, does not further depend on *B*. Note that *A* is marginally independent of *B* when *C* is empty, denoted as $A \,\perp \!\!\!\perp \, B$. Our model equations and Fig. 1 are consistent with the following two conditions:

1. $$\textbf{Z} \,\perp \!\!\!\perp \, U$$: confounder independence
2. $$Y \,\perp \!\!\!\perp \, \textbf{Z} \mid (X,U)$$: exclusion-restriction.

Condition 1 states that each of the instrumental variables included in $\textbf{Z}$ is independent of *U*. This condition is utterly untestable, due to *U* being unobserved. Condition 2 states that there is no association between $\textbf{Z}$ and *Y* other than that mediated by *X*, and can be at best only partially tested. An additional condition is that association between each IV in $\textbf{Z}$ and *X* is not null. This is not strictly required for identifiability, as the model is identifiable when only one component of $\textbf{Z}$ is associated with *X*, and is motivated by our concern that in the presence of a small sample a multitude of irrelevant instruments may create a bias-inducing association between $\textbf{Z}$ and *U*, thereby violating one of the above assumptions.

Prior specifications required to complete the Bayesian formulation of the model are discussed at length in Berzuini et al. [13]. In particular, we shall hereafter take β to be independent from the remaining parameters in the prior. In our simulations, we have taken $\sigma _{X}$ to follow a priori an inverse-gamma distribution, $\sigma _{X} \sim$ *Inv-Gamma*(3, 2), and each component of $\varvec{\alpha }$ to be independently normally distributed with mean 0.5 and standard deviation 0.2:

$$\begin{aligned} \varvec{\alpha } = \left( \begin{array}{c} \alpha _{1} \\ \alpha _{2} \\ \vdots \\ \alpha _{J} \\ \end{array}\right) \sim N\left[ \left( \begin{array}{c} 0.5 \\ 0.5 \\ \vdots \\ 0.5 \\ \end{array}\right) ,\left( \begin{array}{cccc} {0.2}^{2} &{} 0 &{} \cdots &{} 0 \\ 0 &{} {0.2}^{2} &{} \cdots &{} 0 \\ \vdots &{} \vdots &{} \ddots &{} \vdots \\ 0 &{} 0 &{} \cdots &{} {0.2}^{2} \\ \end{array}\right) \right] . \end{aligned}$$

The prior for $\beta$ will be discussed in the Causal discovery decision rule based on ROPE posterior odds and Importance sampling calculation of the posterior odds sections.

### Region of practical equivalence (ROPE)

Having specified the model Eqs. (1-3), one would often define a “point” null hypothesis $\beta = 0$. As previously mentioned, this choice has drawbacks. One of them is that with this choice the subspace of data generating processes corresponding to a “non-existent” causal effect, ie $\beta = 0$, is singular with respect to the full space of data generating processes defined by the model, so that if we fit a model with a continuous prior for $\beta$, the posterior probability of a nonexistent causal effect will be zero. When inference computations are performed via Markov chain Monte Carlo (MCMC) simulation (Metropolis et al. [16]), the probability of the chain visiting the point-space $\beta =0$ will, in this case, be zero, which prevents us from calculating and comparing posterior probabilities for the “non-existence” and for “existence” hypotheses on the basis of the MCMC-generated samples. This problem disappears if we define an interval-valued null hypothesis, that is, by allowing the user to specify a real positive *T*, and:

$$\begin{aligned} H_{0}:-T\le \beta \le T, \end{aligned}$$

$$\begin{aligned} H_{1}:\beta \notin [-T,T], \end{aligned}$$

where without loss of generality we have taken the $[-T,T]$ interval, called ROPE, to be symmetric with respect to zero. With this choice, if we assign an appropriate prior distribution to β (and to the remaining model parameters) then the proportion of times the MCMC chain visits the ROPE will provide us with a simulation-consistent estimate of the posterior probability for $H_0$, as a basis for the hypothesis test decision. We shall claim a “discovery” when the posterior probability of $\beta$ falling in the ROPE does not exceed a specified threshold. In the Importance sampling calculation of the posterior odds section, $\beta$ is given a mixture prior that allows the user to assign a prior probability mass for $\beta$ falling within the ROPE, ie, for the null causal hypothesis.

The value of *T* should in principle be chosen in such a way that ROPE contains values of $\beta$ that have small enough absolute magnitudes to be devoid of practical relevance (Kruschke [5]). This may be straightforward if we are able to come up with a realistic range of values for the causal effect $\beta$. One such situation occurs when the outcome model is a logistic regression, as in Eq. 3. This is, for example, the case in our illustrative study, where the exposure variable, body mass index (BMI), has been standardised to have unit variance, and $\beta$ represents the coefficient of this variable in a logistic regression model of the binary outcome, with no effect interactions on a multiplicative scale. In such a setting, biological insight will suggest a realistic range of values for $\beta$. For example in our illustrative study, where $\beta$ represents the causal effect of standardised BMI on occurrence of juvenile myocardial infarction (JMI), a value of, say, $\beta =0.1$ implies that a one-standard-deviation change in BMI moves the probability of JMI from 0.993 to 0.994, or from 0.524 to 0.549, or from 0.109 to 0.119. In this case we may be willing to judge these changes “negligible” and set $T=0.1$. There is always, of course, the possibility to assess the sensitivity of the results to changes in *T*. In the Simulation experiment section, we *calibrate* *T* with respect to the data and to the model prior distribution. The use of an interval null hypothesis is intended to attenuate the risk of artefactually significant estimates of the causal effect.

### Causal discovery decision rule based on ROPE posterior odds

 Once the model has been specified according to Eqs. (1-3), complete with a prior specification for $\beta$ and the remaining unknown parameters and for the prior probability of $H_0$ being true (details given later) we would run MCMC (see Betancourt [17]) in the space of the unknown model parameters, and then focus on the posterior samples for $\beta$. Relevant quantities are the proportion $V_1$ of sampled values of $\beta$ falling outside the ROPE, and the proportion $V_0$ of values falling inside the ROPE. The $\frac{V_{0}}{V_{1}}$ ratio provides a simulation consistent approximation to the posterior odds of $\beta$ falling inside the ROPE and of the Bayes factor for $H_{0}$ vs $H_{1}$, as a basis for taking the test decision, for example in accord with the following ternary *Causal Discovery Decision Rule* (CDDR) (Schönbrodt and Wagenmakers [18]):

- If $\frac{V_{0}}{V_{1}} > 10$, accept the non-existence (of the causal effect) hypothesis with confidence;
- If $\frac{V_{0}}{V_{1}} < 0.1$, accept the existence hypothesis (and claim a causal discovery) with confidence;
- If $0.1 \le \frac{V_{0}}{V_{1}} \le 10$, conclude in favour of an *uncertain evidence* of a causal effect (uncertain outcome of the decision).

Our chosen thresholds (0.1, 10) are not carved in stone. Not unlike the nominal threshold for the frequentist *p*-value, they will generally depend on the context. Our provision of an “uncertain” outcome of the decision will later allow us to construct a (loss function based) measure of performance of the decision rule that captures the difference between the loss due to rejecting a true hypothesis in favour of “uncertain outcome” and the loss due to accepting a false hypothesis.

Let us now discuss the prior distribution for the causal effect magnitude $\beta$. As mentioned previously, one possibility is to assign $\beta$ a mixture prior distribution, that puts a $\pi _0$ probability on $\beta$ falling in the ROPE according to a rectangular distribution, and a complementary, $(1-\pi _0)$, probability on this parameter being drawn from a locally uninformative continuous distribution that we, without loss of generality, assume to be normal. The parameter $\pi _0$ can be interpreted as the prior probability for $H_0$, with $\pi _0=0.5$ expressing prior ignorance about the existence of a causal effect. Our previous considerations about the choice of the threshold *T* are relevant here. Details about the proposed inference computation procedure are given in the next subsection.

### Importance sampling calculation of the posterior odds

The posterior samples of $\beta$ can be conveniently generated by using the following *mixture prior resampling* scheme. Let $\theta$ denote the full set of unknown quantities in the model (including parameters and missing data values) except the causal effect $\beta$. The idea is to assume prior independence of $\beta$ and $\theta$ , and then apply MCMC to a model where the “true” (mixture) prior for $\beta$, which we denote as $p^{true}(\beta )$, is replaced by a (computationally more convenient) continuous prior denoted as $p^{used}(\beta )$. A sample *S* of values of $(\beta ,\theta )$ will thus be generated from the “incorrect” posterior distribution

4 $$\begin{aligned} \pi ^{used}(\beta ,\theta \mid D) \;\; \propto \;\;p(D \mid \beta ,\theta ) \;\; p^{used}(\beta) \;\; p(\theta ) \end{aligned}$$

where the symbol $\propto$ stands for “proportional to” and *D* denotes the data.

The idea is then to exploit principles of importance sampling in order to correct for the fact that we are sampling (4) instead of the correct posterior. This is described in the following.

The *true* posterior probability for $(\beta ,\theta )$ is given, up to a proportionality constant we do not need to compute, by

$$\begin{aligned} \pi ^{true}(\beta ,\theta \mid D)\propto p(D \mid \beta ,\theta ) \; p^{true}(\beta) \;\; p(\theta ). \end{aligned}$$

${\rm which}$ can be re-written as

5 $$\begin{aligned} \pi ^{true}(\beta ,\theta \mid D)&\propto p(D \mid \beta ,\theta ) \;\; p^{used}(\beta) \;\; p(\theta ) \;\;\frac{p^{true}(\beta )}{p^{used}(\beta )}\nonumber \\&\propto \pi ^{used}(\beta ,\theta \mid D) \;\;\; \omega (\beta ), \end{aligned}$$

where

$$\begin{aligned} \omega (\beta ) \equiv \frac{p^{true}(\beta )}{p^{used}(\beta )}. \end{aligned}$$

Let *S* denote a set of *K* samples of $(\beta ,\theta )$,

$$\begin{aligned} S\equiv \left\{\beta ^{[k]},\theta ^{[k]}\right\},\qquad \qquad k=1,\ldots ,K, \end{aligned}$$

generated from the “incorrect” posterior distribution $\pi ^{used}(\beta ,\theta \mid D)$. A reweighted resampling of *S* with replacement, with the weight of each *k*th sample, $\omega ^{[k]}$, obtained by evaluating $\omega (\beta )$ at it, $\omega ^{[k]} \equiv \omega \left(\beta ^{[k]}\right)$, will , by virtue of (5), yield a set of samples of $(\beta ,\theta )$ we can think of as generated from the correct posterior. In particular, it will yield a set of posterior samples for $\beta$ which we can use according to the CDDR of the preceding subsection to determine the test decision outcome.

Let $p^{true}(\beta)$ take the form of a mixture of a locally uninformative normal distribution, say $N(0,10^{2})$, and a uniform density over $[-T,T]$. ${\rm Let}$ ${\rm then \; the \; "incorrect" \; prior \; for}$ $\beta$, which we have denoted as $p^{used}(\beta )$, be $N(0,10^{2})$. Then:

$$\begin{aligned} \omega ^{[k]}=\frac{(1-\pi _0)\cdot N(\beta ^{[k]} \mid 0,10^{2})+\pi _0\cdot Unif\left(\beta ^{[k]} \mid -T,T\right)}{N\left(\beta ^{[k]} \mid 0,10^{2}\right)}. \end{aligned}$$

where $Unif(q\mid -T,T)$ denotes the probability density at a real point *q* under a rectangular (uniform) distribution with support $(-T, T)$. For a sample $\beta ^{[k]}$ falling outside $[-T,T]$ this weight will be 0.5. For a sample $\beta ^{[k]}$ falling inside $[-T,T]$ and with $\pi _0=0.5$ it will be $0.5 + \frac{1}{4\cdot T\cdot N(\beta ^{[k]} \mid 0,10^{2})}$. Hence samples falling outside the interval will be downweighted with respect to the samples inside, the downweighting being the more pronounced the smaller the interval.

### Calibration

Hypothesis test decision procedures should be evaluated, more precisely *calibrated*, in accord with the principles of decision-theory, by using some measure of the *expected loss*. We shall consider *ternary* decisions with possible outcomes “accept the hypothesis $H_1$ of existence of the causal effect”, “accept the hypothesis $H_0$ of absence of the causal effect” and “uncertain outcome”. Let $L(\beta , A)$ denote the loss incurred when the true value of the causal parameter is $\beta$ and the chosen decision outcome is *A*. Let this function be defined as

$$\begin{aligned} \begin{array}{lr} L(\beta =0, H_{0}\;\text {accepted with confidence})\quad &{}= 0,\\ L(\beta =0, \text {uncertain outcome})\quad &{}= a,\\ L(\beta =0, H_{1}\;\text {accepted with confidence})\quad &{}= 1,\\ L(\beta = \beta ^{*}, H_{0}\;\text {accepted with confidence})\quad &{}= 1,\\ L(\beta = \beta ^{*}, \text {uncertain outcome})\quad &{}=a,\\ L(\beta = \beta ^{*}, H_{1}\;\text {accepted with confidence})\quad &{}=0, \end{array} \end{aligned}$$

where $$\beta ^{*} \notin \; [-T, T ]$$.

The choice of *a*, with $0 \le a \le 1$, will depend on the applicative context. Large values of *a* will be appropriate if more conservative discoveries are desired at the cost of more decisions being held in a limbo.

The next section describes a simulation experiment where we compare results of a frequentist and of a Bayesian MR analysis of the same data, by letting the priors vary during the experiment. In each of the simulated scenarios, the comparison is based on the expected loss:

$$\begin{aligned} Expected \;L{} & {} = p(\text {uncertain outcome}\mid \beta =0) \times \textrm{I}_{\beta =0}\times a \\{} & {} \quad + p(H_{1}\;\text {accepted with confidence} \mid \beta =0)\times \textrm{I}_{\beta =0}\\{} & {} \quad + p(H_{0}\;\text {accepted with confidence} \mid \beta = \beta ^{*}) \times \textrm{I}_{\beta =\beta ^{*}}\\{} & {} \quad + p(\text {uncertain outcome} \mid \beta = \beta ^{*})\times \; \textrm{I}_{\beta =\beta ^{*} } \;\times a. \end{aligned}$$

with the probabilities $p(.\mid \beta =.)$ estimated by simulation. The first and last terms in the expression of *L* will be zero in the frequentist case. The intention in the experiment will by no means be to prove that one of the two inference paradigms is superior, but rather that use of a Bayesian approach with a ternary decision rule might often be a good idea, and that one reason for this is that the frequentist method (but not the Bayesian one) is generally “handicapped” by its inability to allow for an uncertain outcome.

## Simulation experiment

We are now going to describe a simulation experiment where the expected loss criterion of the preceding section is used to compare performances of a frequentist and of a Bayesian MR method in realistic data analysis scenarios. By allowing for randomly missing values of the exposure variable, we shall factor into the experiment the ability of the Bayesian approach to coherently handle missing data, as discussed elsewhere (Zou et al. [14]). This is motivated by the fact that certain MR analysis situations, e.g. involving overlapping samples (see Zou et al. [14]), may be characterized by a high proportion of missing data values.

### Experiment design

We simulated situations where the following two datasets are jointly analysed:

- Dataset *A*: collected from sample individuals with complete observations for $(\textbf{Z}, X, Y)$;
- Dataset *B*: collected from individuals with completely observed values for $\textbf{Z}$ and *Y*, and completely missing values of *X*.

We assumed no overlap, i.e., no individuals shared between *A* and *B*. Let the symbol $D_1$ denote the combined dataset $A \cup B$. In the special case where *B* is empty, dataset $D_1$ lends itself to standard one-sample MR analysis. Analysis of $D_1$ will otherwise fall in the “one-sample MR with missing *X*-data” category. With reference to Eqs. (1)-(3), we considered 18 different configurations:

- the rate of missingness of *X*: (80%, 40%, 0%)
- the strength of the $\varvec{\alpha } = (\alpha _{1}, \alpha _{2}, \ldots , \alpha _{J})$ coefficients, assumed to be the same for all IVs: $(\mathbf {0.3}$, $\mathbf {0.1}$, $\mathbf {0.05})$
- the magnitude of the causal effect $\beta$: (0.3, 0)

In total, $3 \times 3 \times 2 = 18$ scenarios were simulated. Throughout the experiment, parameters $\delta _{X}$ and $\delta _{Y}$ were set to 1 and the number *J* of instruments was set to 15. Two hundred datasets were simulated under each separate scenario, for a total of 3,600 datasets simulated during the experiment. Each of these 3,600 datasets was generated by the following sequence of steps:

1. simulate 1000 independent individuals characterized by realistic realizations of $\textbf{Z}$ and then, on the basis of the *Z*s, generate for each individual values of *X* and *Y* in accord with Eqs. (1)-(3). Call the resulting dataset ${\textbf {H}}$;
2. randomly sample $n_A$ individuals from ${\textbf {H}}$, without replacement, and let the selected individuals, each with a completely observed ($\textbf{Z}, X, Y$) vector, form the dataset that we have previously labelled as *A*;
3. randomly sample $n_B$ individuals from ${\textbf {H}} \setminus A$ and take each of them to be characterized by observed ($\textbf{Z}, Y$), with their corresponding values of *X* treated as missing. Let these selected individuals form the dataset that we have previously labelled as *B*. At this point, we were ready to apply MR to data $D_1 = A \bigcup B$.

The sample size of $D_1$ was set to be 400 throughout the experiment. Parameter $n_B$ was controlled by the rate of missingness of *X* for the relevant scenario. For example, for a rate of missingness of *X* of 80%, we had $n_A = 80$ and $n_B = 320$. In the special case of a 0% rate of missingness of *X*, it was $D_1 \equiv A$. MCMC computations were performed with the aid of the probabilistic programming language Stan that also incorporates variational inference methods (Stan Development Team [19, 20]). Missing data imputation and causal effect estimation were performed simultaneously via MCMC, by exploiting the substantial equivalence of unknown model parameters and missing values in Bayesian analysis.

We compared our Bayesian method with the following frequentist approach to two-sample MR: inverse-variance weighted (IVW) estimation (Burgess and Thompson [21], Bowden et al. [22]). Application of IVW required the observed values of *Y* in Dataset *A* to be discarded to comply with the frequentist two-sample analysis mechanism. After each frequentist MR analysis of a simulated dataset, the null causal hypothesis $H_{0}:\beta =0$ was accepted iff the 95% confidence interval for $\beta$ contained the null value 0. The alternative hypothesis $H_{1}:\beta \ne 0$ was otherwise accepted.

As far as the Bayesian analysis of each simulated dataset is concerned, we used the previously discussed model and computational procedure and CDDR decision rule to determine the outcome of the test. At each new simulation run, the threshold *T* and parameter *a* were randomly drawn from uniform distributions $T \sim Unif\left(10^{-2}, 10^{-1}\right)$ and $a \sim Unif(0, 0.6)$ in order to assess sensitivity of performance to changes in these parameters. The information generated by the above procedure allowed us to compute the expected losses for the MR methods under comparison, a high expected loss implying a high rate of false positives/negatives.

### Experiment results

**Fig. 2:**
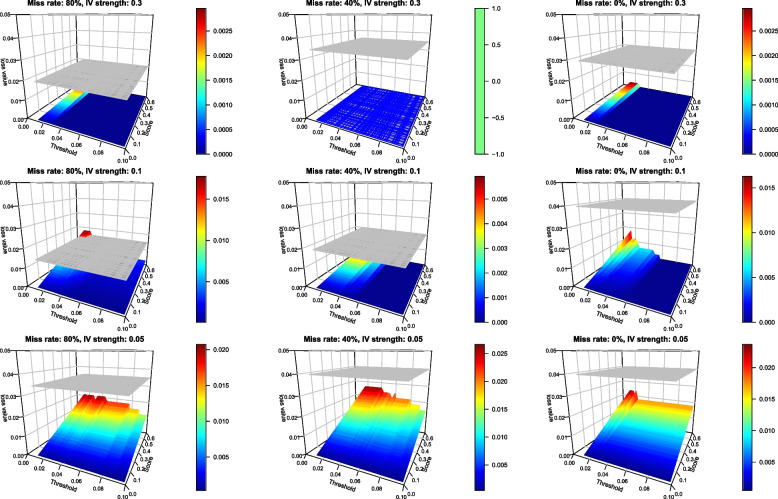
Loss of the frequentist (grey) and our Bayesian MR with binary *Y* when $\beta = 0$ for different combinations of missing rate of *X* (80%, 40% and 0%) and IV strength (0.3, 0.1 and 0.05), based on 200 simulated datasets per combination. Loss is pictured for different values of the threshold, *T*, and of the score *a*

Figure 2 displays the results, when $\beta = 0$, of the frequentist (IVW) and Bayesian MR for each combination of IV strength and missing rate. The flat grey surface in each panel depicts the loss of the frequentist MR and the coloured surface the loss of the Bayesian method. Unsurprisingly, the average loss incurred by the frequentist approach does not appear to depend on the values of *T* or *a*, as these parameters are not involved in the frequentist decision rule.

Our Bayesian method showed a lower loss almost uniformly across the configurations, with the average loss decreasing with an increase in IV strength. There were noticeable fluctuations of the loss when *T* was small. This is due to calculation of the posterior odds, as estimated by $\frac{V_0}{V_1},$ becoming rather unstable when the number of samples of $\beta$ falling in $[-T,T]$ becomes small.

**Fig. 3:**
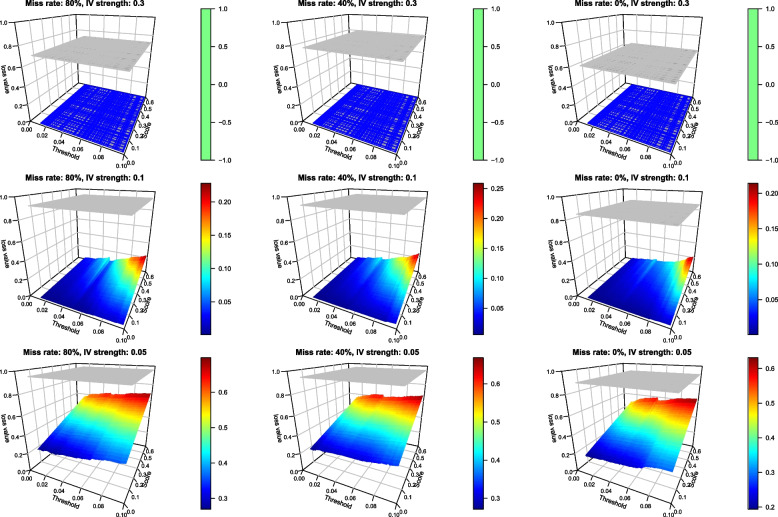
Loss of the frequentist (grey flat surfaces) and our Bayesian MR (coloured curves) with binary *Y* when $\beta = 0.3$ (outside the tolerance interval). Panels represent different combinations of the missing rate of *X* ($80\%,$ $40\%$ and $0\%$) and the IV strength (0.3, 0.1 and 0.05). 200 datasets were simulated for each of these scenarios

When $\beta = 0.3$, our Bayesian method resulted in no loss when $\varvec{\alpha } = \mathbf {0.3}$ (Fig. 3), showing a positive impact of a higher IV strength on decision performance. This is because no posterior samples fell in $[-T,T]$ for all different values of *T*. As IV strength decreased, the posterior distribution had a higher spread and some samples fell in the tolerance interval for a large *T*, and we started to see a loss from the Bayesian method. When *T* continued to increase, the wider tolerance interval went on receiving more samples, leading to a higher loss. When the level of IV strength decreased, the loss increased in our method. However, our method still performed consistently better than the frequentist.

## Is obesity a strong cause of juvenile myocardial infarction?

With the improvement of living standards, recent decades have witnessed a dramatic increase in prevalence of obesity. A common measure of obesity is the body mass index (BMI), defined as the weight in kilograms divided by the square of the height in meters. A high value of BMI is taken to indicate an excess of body fat. Studies have demonstrated that obesity acts as a major causal risk factor for cardiovascular disease and hypertension. To the best of our knowledge, no studies have examined these links that focuses on occurrence of MI *at an early age*, sometimes called *juvenile myocardial infarction* (JMI). Yet, early age is where the influence of genetics on cardiovascular events is at its highest, which makes a MR analysis of the causes of early-age MI most appealing and less vulnerable to biases. Motivated by these considerations, our study focuses on the causal effect of BMI on occurrence of a MI before age 45.

Our analysis was based on data from an Italian study of the genetics of infarction (Berzuini et al. [23]). JMI cases were ascertained on the basis of hospitalization for acute myocardial infarction between ages 40 and 45, between calendar years 1996 and 2002. The recorded values of BMI, measured after occurrence of JMI, were considered representative of pre-JMI obesity level.

One caveat in the analysis we are going to describe is that post-JMI BMI levels may reflect recent changes in the patient’s lifestyle and behaviour, resulting in rather weak genetic associations with BMI and, as a consequence, in higher vulnerability to bias.

**Fig. 4:**
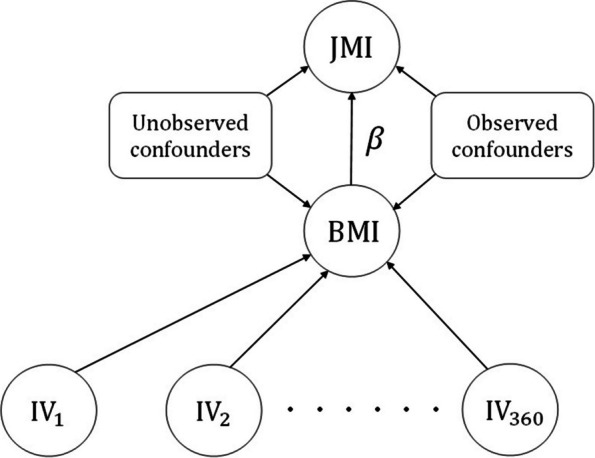
MR model with 360 instrumental variables, ($\mathrm IV_{1}$, $\mathrm IV_{2}$, ..., $\mathrm IV_{360}$), used in our illustrative study to assess the causal effect of BMI on juvenile myocardial infarction (JMI). Observed values of 5 potential confounders (sex, smoking status, alcohol consumption, cocaine consumption and age), were included in the model (see main text). The model assumes there are no associations between IVs and JMI other than those mediated by BMI

We adopted the model described by Eqs. (1)-(3), with uninformative priors with respect to the parameter region of posterior importance. This was characterized by a logistic regression dependence of the binary outcome on the exposure, as in the referenced equations, conditional on the following potential confounders: sex (Male/Female), smoking status (Yes/No), alcohol consumption (Yes/No), cocaine consumption (Yes/No) and age. A graphical representation of the model is shown in Fig. 4. The narrow age range of the event in our study attenuates problems introduced by censoring, and justifies our choice of representing disease outcome as a binary variable (1: had MI, 0: did not have MI) in our analysis.

The single nucleotide polymorphisms (SNPs) associated with BMI were identified based on the datasets from genome-wide association study (GWAS) of the UK Biobank^1^ ($P\le 5\times 10^{-8}$). Through the command-line program Plink (Purcell and Chang [24]), as many as 360 independent ($r^{2} < 0.001$) SNPs were selected and then used as instruments for assessment of obesity causal effect on JMI. SNPs were coded as 3 valued (0, 1, 2) counts of the minor allele, after appropriate cross-study harmonization. In total, 521 independent individuals were studied in the illustrative analysis based on our Bayesian method. Values of BMI were standardised (mean 0, standard deviation 1) prior to analysis.

**Fig. 5:**
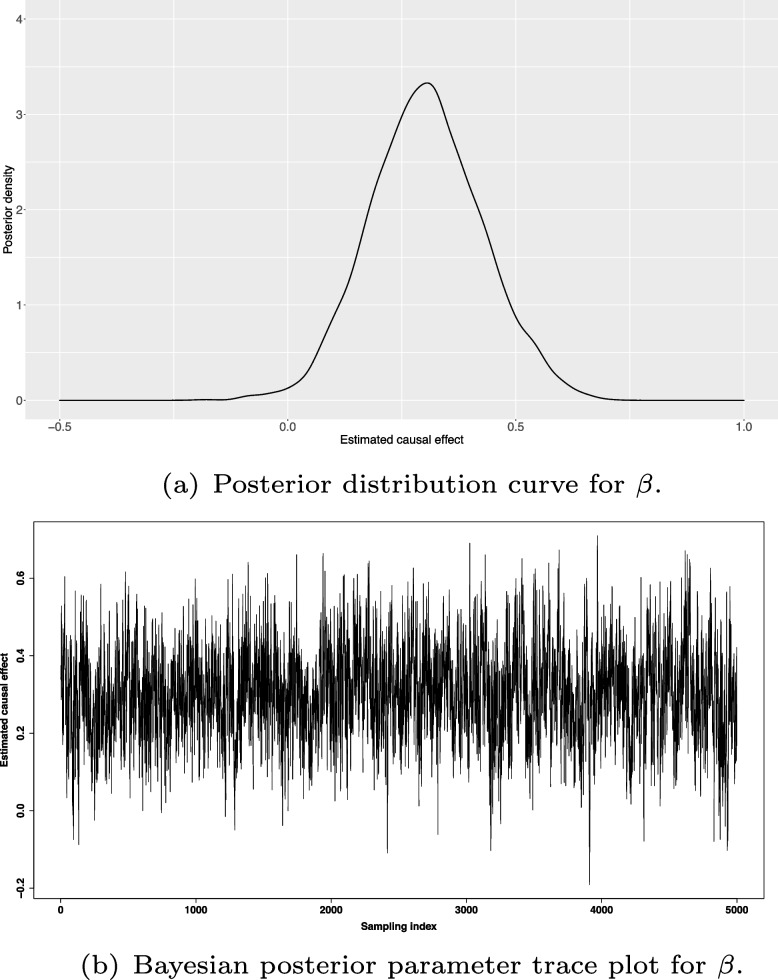
Estimated causal effect of *standardized* BMI on JMI, based on the Bayesian Mendelian randomization analysis we have performed data from our illustrative study. (*a*) Posterior distribution curve for causal parameter $\beta$ with posterior mean 0.303 and 95% credible interval (0.069, 0.550). (*b*) Bayesian posterior parameter trace plot for $\beta$

We ran the Markov chain in the space of the model unknowns (model parameters and missing BMI values). The chain was 20,000 iterations long, the last 5,000 iterations being used for purposes of inference. Figure 5 shows posterior density plot (Panel (a)) and Bayesian posterior parameter trace plot (Panel (b)) for the causal effect of standardized BMI on JMI. The trace in (b) indicates reasonably good mixing of the chain, with $\widehat{R}=1.002$. For each of the thresholds (0.02, 0.04, 0.06, 0.08) for *T*, after the *mixture prior resampling* discussed in Importance sampling calculation of the posterior odds section, we had $0.1< \frac{V_{0}}{V_{1}} < 10$. In spite of the 95% credible interval for $\beta$ lying entirely in the positive real axis, we therefore conclude in favour of *uncertain evidence* of a causal effect of genetically induced changes in obesity on JMI.

## Discussion

In our approach to Bayesian MR analysis, the hypothesis $H_0$ of *non-existence* of the causal effect of interest is represented by a user-specified interval of values of the causal effect, which we may refer to by using the established term “region of practical equivalence” (ROPE). Importance sampling technology is used to approximate the posterior odds of the causal effect falling inside this interval. A sufficiently large value of this posterior odds will lead to acceptance of the null no-effect hypothesis, whereas a sufficiently small value will lead to acceptance of the alternative hypothesis, and to a causal discovery claim. A third, “uncertain”, decision outcome is available for situations where the posterior odds is neither large nor small enough, indicating scarce data support to either hypothesis. The uncertain outcome has been introduced to reduce chances of placing undue confidence in a hypothesis that is only weakly supported from the data. We have incorporated this ternary test decision logic into the Bayesian MR framework proposed by Berzuini et al. [13] and further refined by Zou et al. [14]. The decision rule can be calibrated via simulation by acting on the differential weighting parameters of a loss function.

In a simulation experiment, we have compared our method with a standard MR method in terms of expected loss, by allowing loss function parameters to vary within reasonably wide intervals. The experiment suggests that, within the examined scenarios, our method outperforms standard MR, and this may be due to the latter being handicapped by inability to accommodate decision uncertainty. We consider our proposed method as a contribution to research on more reproducible MR analysis.

We have applied our proposed method to a MR study of the causal effect of obesity on *juvenile* myocardial infarction, based on a unique dataset. The study concludes in favour of an *uncertain evidence* of a non-null causal effect.

## Data Availability

The data of simulation experiment and illustrative study is available from the corresponding author upon request.

## Footnotes

1 We use the datasets from https://www.ebi.ac.uk/gwas/publications/25673413 and http://biobank.ctsu.ox.ac.uk/crystal/field.cgi?id=21001.

## Code availability

The code of data simulations and illustrative study is available from the corresponding author upon request.
